# Nutritional Rehabilitation: Practical Guidelines for Refeeding the Anorectic Patient

**DOI:** 10.1155/2010/625782

**Published:** 2010-02-07

**Authors:** Philip S. Mehler, Amy B. Winkelman, Debbie M. Andersen, Jennifer L. Gaudiani

**Affiliations:** ^1^Department of Internal Medicine, Denver Health Medical Center, Denver, CO 80204, USA; ^2^Food and Nutrition Services Department, Denver Health Medical Center, Denver, CO 80204, USA

## Abstract

Weight restoration is crucial for successful treatment of anorexia nervosa. Without it, patients may face serious or even fatal medical complications of severe starvation. However, the process of nutritional rehabilitation can also be risky to the patient. The refeeding syndrome, a problem of electrolyte and fluid shifts, can cause permanent disability or even death. It is essential to identify at-risk patients, to monitor them carefully, and to initiate a nutritional rehabilitation program that aims to avoid the refeeding syndrome. A judicious, slow initiation of caloric intake, requires daily management to respond to entities such as liver inflammation and hypoglycemia that can complicate the body's conversion from a catabolic to an anabolic state. In addition, nutritional rehabilitation should take into account clinical characteristics unique to these patients, such as gastroparesis and slowed colonic transit, so that measures can be taken to ameliorate the physical discomforts of weight restoration. Adjunct methods of refeeding such as the use of enteral or parenteral nutrition may play a small but important role in a select patient group who cannot tolerate oral nutritional rehabilitation alone.

## 1. Background

Refeeding the anorectic patient is essential to achieving a successful treatment result. Judging by outcome studies from around the world, most experts agree that one cannot effectively treat anorexia nervosa without first restoring body weight. It is also clear that without a concerted refeeding effort, no meaningful psychotherapy can take place, due to starvation-induced cognitive deficits. However, weight restoration may be one of the most challenging and frustrating parts of the recovery process for many patients with anorexia nervosa. 

 Traditionally, centers that treat patients who have moderate and severe degrees of anorexia nervosa have used a combination of behavioral techniques, cognitive restructuring, and a progressive structured program of oral caloric intake, to achieve the goal of weight restoration. Different types of gastric feeding and total parenteral nutrition (TPN) may be indicated on a rare basis for more refractory cases. TPN is a specialized procedure and should be undertaken only when medically necessary and by an experienced clinician, with the support of an experienced nursing and nutritional staff. Clinicians caring for these patients must be cognizant of the art of the refeeding process, given the multitude of potential clinical and biochemical caveats that can develop. 

 In general, the nutritional needs and goals of anorectic individuals are based on attaining a healthy IBW. For a female, IBW may be calculated as follows: 100 pounds for five feet of height, and five pounds for each inch greater than five feet tall. For a male, the IBW may be calculated as follows: 106 pounds for five feet of height, and six pounds for each inch thereafter. While these formulae are older and do not reflect patients' body compartments, ethnicity, and age, they remain a reasonable estimate of appropriate body weight. As such, the calculated ideal body weight remains the academic standard by which patients' degree of underweight is identified. Patients can be classified as mild, moderate, severe, or critical based on whether they are 10 percent, 20 percent, 30 percent, or more, respectively, below IBW. An alternative way of classifying patients is based on body mass index (BMI), which is calculated based on height and weight and is readily available in tables for an array of heights and weights. Specifically, the BMI value is obtained by dividing the patient's weight in kilograms by their height in meters squared. It is however limited in that it does not provide any measure of body composition or of nutritional status [1]. Of note, standardized tables of “ideal body weight” may not be appropriate for use with adolescents. Normative data from the National Center for Health Statistics height and weight tables may be better suited for adolescents 12 to 18 years of age. 

 When a patient is set to begin the refeeding process, there must be, from the onset, an earnest attempt to achieve agreement among the caregiver, dietitian, and patient as to what the target weight is going to be. Also, spending time educating a patient with anorexia nervosa about metabolism and how it may change during the process of weight restoration may prevent future difficulties and reduce stress for the patient. Generally, weight gain to within ten percent of IBW is an acceptable goal, regardless of the mode of refeeding. Some view a “healthy weight” as being the weight at which normal menstruation occurred in the past. However, if amenorrhea persists, it may be necessary to actually achieve IBW, or even a little above it. Discharging patients before they reach a minimum normal weight is associated with an increased rate of readmission [2, 3]. Many practitioners will admit a patient to the hospital for inpatient treatment and nutritional restoration when the patient's weight is more than 30 percent below IBW. This is both to minimize morbidity during the early stages of refeeding and because the rate of severe complications seems to markedly increase at these low weights.

## 2. Refeeding Syndrome

Before describing the specifics of refeeding, it is prudent to describe a potentially catastrophic complication that can occur during this process. It is referred to as the refeeding syndrome. This syndrome occurs in significantly malnourished patients during the early phase of nutritional replenishment whether it is by the oral, enteral, or parenteral route. The new National Institute for Health and Clinical Excellence (NICE) Guidelines for Management of Refeeding Syndrome, released this year, establish two sets of criteria for identifying patients at high risk for refeeding syndrome. (Box 1) [4] The risk of the refeeding syndrome is directly correlated with the degree of weight loss which has occurred as a result of the anorexia nervosa. Thus, it is self-evident why patients who are more than 30% below their IBW should be initially refed during an inpatient hospitalization [5, 6]. Earlier in this century, the heart was believed to be immune to the effects of chronic malnutrition. However, during World War II, experiments were performed using conscientious objectors who voluntarily agreed to lose a certain percentage of their body weight. As a result of this weight loss, low blood pressure developed and cardiac size, as seen on a simple chest radiograph, diminished. This led to the realization that the heart can be adversely affected by weight loss as well as subsequent refeeding. 

 The mechanism of the potential cardiovascular collapse that occurs with the refeeding syndrome is multifactorial. First, the reduced heart mass that accompanies weight loss makes it difficult for the heart to handle the increase in total circulatory blood volume seen with refeeding. The end result of this can be heart failure. Even though the heart mass does revert toward normal with weight gain, the first few weeks of refeeding require close attention to an anorectic patient's cardiovascular status until this normalization process has occurred. Studies in anorexia nervosa have shown diminished cardiac output as a result of atrophy of the heart muscle which accompanies unhealthy weight loss [7]. 

 Second, changes in serum levels of phosphorous as well as potassium and magnesium are key variables in the refeeding syndrome. The mechanism by which a low phosphorous level (hypophosphatemia) develops during refeeding is mainly due to the glucose content of the food substrate. The glucose load increases insulin release which in turn produces shifts of phosphate and potassium into the intracellular space. There is also an incorporation of phosphate into newly synthesized tissues during refeeding. The resultant low phosphate levels are accompanied by depletion of the high-energy chemical, adenosine triphosphate (ATP), which impairs the contractile properties of the heart, and can evolve into congestive heart failure. Refeeding-induced hypophosphatemia can also result in diaphragmatic muscle fatigue and respiratory failure. Other sequealae of refeeding-induced hypophosphatemia include red and white blood cell dysfunction, skeletal muscle injury (rhabdomyolysis) and seizures. Rhabdomyolysis is diagnosed by finding an abnormally high level of the muscle enzyme, creatinine phosphokinase (CPK), on a simple blood test. Low levels of serum potassium and magnesium can also cause cardiac irritability and arrhythmias along with skeletal muscle weakness [8].

## 3. Practical Tips for Refeeding

This medically worrisome refeeding syndrome is usually preventable. The general dictum to follow with regard to dietary calorie repletion is “Start low, advance slow.” The caloric requirements for the female anorectic patient can be accurately calculated with the Harris Benedict equation [BEE = 6.55 + (9.6 × body weight in kg) + (1.8 × height in cm) − (4.7 × age in years)]. Dietitians are most familiar with this calculation. Or, the basal energy expenditure (BEE) can be measured by indirect calorimetry, a relatively simple procedure involving a breathing test. It is available in many hospitals through their nutrition department. Calorimetry is based on measurement of carbon dioxide production and oxygen consumption. The BEE value basically reflects the energy utilized when the body is at rest and in clinical practice the terms BEE and REE are used interchangeably [9]. It must be multiplied by an activity factor (1.2 to 2.0) to determine total energy expenditure (TEE). In general, the TEE exceeds the BEE/REE by 10 to 60 percent, depending on how active the patient is. The TEE should not be the starting point of caloric repletion, due to the risk of the refeeding syndrome. Rather, it is a target to achieve days to weeks after the initiation of refeeding. BEE/REE measured by indirect calorimetry gives a slightly lower value for caloric needs than the values estimated by the aforementioned formula. Therefore, if indirect calorimetry is not obtained to define caloric needs early in the refeeding of these malnourished patients with anorexia nervosa, it may be prudent to err on the low side of estimates derived from the Harris-Benedict formula to avoid the refeeding syndrome. This discrepancy is no longer an issue in predicting BEE/REE approximately three weeks following the beginning of the refeeding period. 

 Although it is clearly important, especially for hospitalized patients, to experience a significant degree of weight gain to maximize recovery, the optimal dietary intervention for effectuating weight gain is unknown. There has been a distinct and surprising paucity of research on nutritional interventions in anorexia nervosa. After the first week or two of an inpatient hospitalization, the usual goal is a 2-3 lb/week rate of weight gain. While some patients arrive at the hospital profoundly volume depleted, others, especially if they have undergone aggressive intravenous repletion or total parenteral refeeding during a recent previous hospitalization, may arrive with as much as twenty extra pounds of edema weight. Even with judicious and slow initiation of caloric intake, the most medically compromised patients may gain some edema weight initially and then will slowly auto-diurese this weight as their true weight begins to rise, while others must lose all the preexisting edema weight before their weight begins to rise in a healthy manner. This can make it difficult to interpret weight change in the first several weeks of hospitalization. 

 Several different approaches have been promoted to minimize the refeeding syndrome and initiate healthy weight restoration. The main tenet is to avoid overly aggressive refeeding protocols early in the refeeding process. Intake levels usually begin at approximately 600–1000 kcal/day and are increased by 300–400 kcal every three to four days. The treatment corollary of this is to individualize intake based on the rate of weight gain. Supplementing the diet with a liquid supplement in the early stages of refeeding to achieve the prescribed calorie goal is a very effective strategy to achieve weight gain. Liquid supplements can also be of value when added to achieve large caloric intake. Some programs recommend ultimately attaining an intake of 4000 to 5000 kcal per day, while others peak at 3000 to 3600 kcal per day. Even for normal weight adults, weight gain does not correlate exactly with the total excess calories ingested over basal requirements. Therefore, it may be difficult to precisely define the factors that consistently correlate with the number calories needed to gain one pound. The basal metabolic rate on admission is invariably low, but it begins to increase early on in the nutritional rehabilitation process. In general, starved anorectics are metabolically inefficient, and may require more than the expected 3,500 kcal beyond maintenance caloric needs to restore a pound of body weight. The caloric requirement necessary to cause weight gain can vary between 1800 kcal/day and 4500 kcal/day. 

 Some simple general rules to follow are: (1) the TEE should never exceed twice the BEE, (2) caloric intake should rarely exceed 70 to 80 kcal per kilogram of body weight or 35 to 40 kcal/lb, (3) with the severely anorectic patient, begin a diet at 20 to 25 kcal per kilogram, (4) protein intake should not exceed 1.5 to 1.7 grams per kilograms of body weight, and is generally in the 1 to 1.5 grams range, (5) if TPN or enteral feedings are being used, carbohydrate intake should not exceed 7 mg/kg/min, (6) weight gain should be in the two to three pounds per week range. Males generally peak at 4,000 kcal/day and females at 3,500 kcal/day. All approaches requires vigilant clinical and laboratory monitoring and individualization of a dietary plan depending on the rate of weight gain and the laboratory and clinical course of the patient. 

 Currently, on most eating disorder units, caloric intake is slowly increased. These increases should always be guided by the patient's actual pattern of weight gain versus the standard goal of two to three pounds per week. Caloric intake must be modified because of changes in the REE during the course of refeeding in anorexia nervosa, which will impact the rate of weight gain. REE is often lower in anorexia nervosa patients early on in the refeeding process than at later stages [10, 11]. It is possible that these changes contribute to the difficulty of ongoing sustained weight gain during the latter stages of refeeding among patients with anorexia nervosa [12]. Caloric requirements for weight restoration in patients with anorexia nervosa are best determined by monitoring an individual's rate of weight gain. Given this dynamic process, caloric requirements may have to be recalculated if weight gain is not being achieved as expected during the refeeding process, or consideration given to potential confounding sources of weight loss, such as covert exercise or other modes of purging. Recalculation of the REE with indirect calorimetry may be reasonable whenever this clinical scenario is manifested. 

 Ultimately, as the process of refeeding progresses and IBW is achieved, very high levels of caloric intake may be temporarily necessary (70 to 80 kcal/kg) to promote ongoing weight gain. It has recently been elucidated that refeeding a patient with anorexia nervosa may be associated with an actual increase in REE during the weight gain process. Although the mechanism of this phenomenon is presently unknown, its clinical implications are quite clear: unusually high-calorie diets may be necessary to provide continued weight gain towards the end of the weight restoration process. A plateau in the desired rate of achievement of a patient's target weight may be observed because of underestimation of caloric needs during the late stages of weight restoration due to the aforementioned change in the REE value. The optimal treatment strategy should be predicated on regularly monitoring an individual's pattern of weight gain and adjusting the dietary plan because of these inherent individual variations. All of these principles apply regardless of whether customary oral feedings are being used or an alternative, less commonly used method, such as total parenteral nutrition (TPN) or enteral feeding, are being used.

## 4. Potential Complications

From a clinical standpoint, rates of weight gain greater than two to three pounds per week are usually not nutritionally sound and may represent edematous fluid retention or retained bowel contents from constipation. Aside from this numeric guide to weight gain, there are clinical parameters that are worthy of being closely followed. Specifically, vital signs contribute useful daily information. Anorectic individuals generally are bradycardic (heart rate <60 beats/minute). Although there are other potential reasons for tachycardia (heart rate >100 beats/minute), the presence of an elevated heart rate, even if just in the 80–90 heart rate range, during refeeding, can be a harbinger of the refeeding syndrome and cardiac compromise. Thus, even if these patients are technically not tachycardic, a sudden sustained increase in the pulse to greater than 80–90 deserves evaluation given anorectic individuals' typical baseline heart rates of 50 to 60 beats per minute. Thus, daily monitoring of vital signs a few times per day is crucial during the first few weeks of the refeeding process. 

 In addition, checking for the presence of edema in the ankle and shin areas is also a worthwhile practice during the early refeeding stages; its presence can be an ominous sign of the refeeding syndrome. However it may also occur as a minor complication because during the early stages of refeeding, insulin secretion normally increases. Insulin induces sodium retention by increasing kidney tubular sodium reabsorption [13]. Low-sodium diets should therefore be utilized as part of the nutritional plan. The presence of rales on pulmonary examination or evidence of elevated jugular venous pressures are uncommon, but if present may imply true cardiac failure. Any of these clinical findings deserves a thorough medical evaluation with consideration given to possibly decreasing the rate of refeeding. In addition, because of the delayed stomach emptying and prolonged colonic transit time found in the severe stages of anorexia nervosa, these patients often complain of abdominal bloating and constipation. A bowel regimen, with a judicious amount of fiber and adequate hydration, may help alleviate these symptoms during the early refeeding process. 

 A multitude of possible fluid and electrolyte aberrations can occur during the refeeding process, especially in the more severe segment of the anorectic population. As the body shifts from a catabolic to an anabolic state, potassium, phosphorous, and magnesium are incorporated into the newly synthesized muscle tissue and are used for intermediary metabolism, which can cause low serum levels, namely, hypokalemia, hypophosphatemia, and hypomagnesemia. As discussed above, phosphorous is the key electrolyte in the refeeding syndrome [14]. Frequent monitoring of the patient's blood chemistry values (potassium, phosphorous, magnesium, sodium, and glucose) can avert these problems. Early in the refeeding process, checking serum chemistry values every day or every other day is a reasonable plan. This can be advanced to biweekly once the patient has consistently gained weight and if their blood profile has remained relatively stable. Ultimately, with continued weight gain and stable laboratory results, this can be further modified to less-frequent monitoring. However, the major emphasis should be on serum phosphorous levels, because refeeding hypophosphatemia is the main chemical cause of the refeeding syndrome. 

 Other metabolic complications might be encountered during the refeeding process. Mild elevations of liver enzymes can be seen during refeeding. They are more common in those patients being refed enterally or with TPN. Generally, the abnormalities are noted after the first few weeks of refeeding. Aspartate aminotransferase (AST) and alanine aminotransferase (ALT) rise first, followed by alkaline phosphatase, and then bilirubin. Usually, these abnormalities have little clinical significance and resolve with a slowing of the rate of the refeeding process. Many hypotheses have been suggested as to the etiology of these abnormalities; much evidence points toward excessive dextrose calories causing fat accumulation in the liver cells which is known as hepatic steatosis. On occasion the elevations can be more pronounced (more than three times normal) and necessitate consultation with a dietitian to reduce carbohydrate-dextrose calories. 

 Thiamine deficiency in the acute refeeding period may cause either congestive heart failure (“wet beriberi”) or Wernicke's encephalopathy (“dry beriberi”), which is characterized by an acute confusional state, with ocular abnormalities, ataxia, hypothermia, and even coma [15, 16]. Central pontine myelinolysis as a complication of refeeding and hyponatremia has also been reported [17]. Atrophy of the intestinal mucosa and pancreatic impairment may cause severe diarrhea during early refeeding [16]. 

 Last, hypoglycemia has been associated with anorexia, related to depleted hepatic glycogen reserves and gluconeogenesis substrates. Large glucose loads contained in aggressive refeeding protocols stimulate substantial amounts of insulin release from the pancreas, which cannot be offset by the depleted hepatic reserves of glycogen. These postprandial insulin surges cause postprandial hypoglycemia. However, patients with extreme malnutrition often additionally have fasting hypoglycemia. Clinicians must always be cognizant of this risk of hypoglycemia early in the course of refeeding, especially if the parenteral or enteral route is being used, and it needs to be abruptly discontinued. Inadvertent or intentional abrupt cessation of TPN or enteral feeds in any patient can result in dangerous hypoglycemia.

## 5. Alternative Modes of Refeeding

Although progressive oral refeeding programs are the basic mode of refeeding a patient with anorexia nervosa, it is worthwhile to discuss alternatives to the traditional oral refeeding mode. The oral refeeding plan, with a strict behavioral protocol, is the first choice of treatment because it provides a less invasive, safer and more therapeutic method of treatment. The dietary goal must be to move from the reduced calorie oriented approach to a truly balanced nutritional program with adequate calories to balance energy expenditures and the patient's developmental stage. There are definite, albeit *infrequent*, indications for TPN and enteral feedings in anorexia nervosa [18]. However, these alternative modes of refeeding are not a panacea for the treatment of anorexia nervosa, and they cannot be recommended as routine therapy for all anorectic individuals. Some potential criteria for their use are: (1) persistent failure to gain weight with other standard dietary therapies; (2) life-threatening weight loss; (3) worsening psychological state despite standard treatments (Box 2). These in part, are based on the fact that the odds of recovery decline as the time spent continuously ill with anorexia nervosa lengthens and thus, on rare occasions these alternative modes of weight restoration might be reasonable.

Controlled studies of nutritional treatments for anorexia nervosa are essentially non existent. Enteral nasogastric feedings (NG) or percutaneous endoscopic gastrostomy-based feeding (PEG) have both been used in refractory cases of anorexia. 

 Both of these enteral modes of nutritional delivery utilize liquid supplements that contain 1–1.5 Kcal/cc. The only difference being whether the tube which delivers the enteral feed is inserted via the nose and down the esophagus into the stomach or through the skin above the stomach and bored into the stomach and secured. These two modes of enteral feeding can either be utilized with a twenty-four hour continuous flow of supplement, or as a bolus feed which takes only ten minutes to push in, with the daily caloric amount divided into two or three rapid feeds. Dietitians are invaluable in calculating the rates and amounts of enteral feeds necessary to achieve weight gain. The advantage of bolus enteral feeds is that the patient is not tied to the pump all day long. The disadvantage is that the bolus mode may cause gastric discomfort and diarrhea and thus be less well tolerated. Anecdotally, although some physicians promote standard use of parenteral or enteral nutrition, [19] patients often find these modalities aversive and some view them as psychologically harmful. Also, patients may complain of gastric fullness with enteral feeds, which is further exacerbated by the delayed gastric emptying that is found in more severe cases of anorexia nervosa. Although nutritionally comparable, these problems are not associated with TPN. However, TPN is more expensive and fraught with catheter-related infection complications. In addition, patients can disconnect and drain TPN, putting them at risk for air embolism. There are no randomized trials to guide the use of TPN versus enteral nutrition in anorexia nervosa. These different modes of nutritional support have been compared in other medical patients and absent unique mitigating circumstances, enteral nutrition is the preferred route given its reduced rate of infectious complications [20]. 

 While standard protocols exist, TPN must be viewed as a drug that is prescribed only for severe and refractory cases with utmost discretion on a very individual basis. It is beyond the scope of this article to discuss all of the specifics. Briefly, with TPN, dextrose and lipid preparations are the main source of energy substrate. This solution is administered on a continuous 24-hour-per-day basis via a catheter, which is either surgically placed in the forearm or in the upper chest and can remain in place for many months. Patients can receive upwards of 3,000 to 4,000 kcal/day via this route in addition to some oral feeds, which must not be interrupted. Although TPN is a powerful tool and has been used successfully in the care of refractory, severely anorectic patients or in anorectic patients who have comorbid gastrointestinal issues which precludes oral caloric intake [21, 22], its administration may be associated with a plethora of mechanical, infectious, and metabolic difficulties. It is therefore best reserved for use in medical centers where expertise in dealing with these potential difficulties exists and for patients who are truly refractory to more traditional refeeding programs. Also, both enteral and parenteral nutrition should rarely supplant the expectation that the patient will resume normal and adequate eating. 

 Similarly, enteral nutrition may infrequently play a role in the treatment of a select group of anorectic patients. It may also play a role as supplemental nocturnal refeeding assist for patients whose rate of weight gain is slow or plateaued [23]. It can be given either via a mercury-tipped nasogastric tube or via a percutaneously placed endoscopic gastrostomy tube. Notwithstanding possible patient aversion to this mode of refeeding, there have been small series of successful outcomes published in the literature using enteral programs. Generally, a polymeric formula is started at a slow rate, with gradual increases in the rate and tonicity of the solution. Potential problems include diarrhea, blockage of the tube, and electrolyte imbalances. Some centers administer it by continuous-drop infusion for 14 hours per day, while others recommend a 24-hour-per-day continuous infusion and some use the aforementioned bolus mode. Hypoglycemia may be a problem during the time when the infusion is stopped when using the 14-hour infusion or bolus protocol especially early in the weight restoration process. Again, given the added potential complications, some level of special expertise is necessary when enteral feeds are considered. There has been debate about the ethics of “involuntary” feeding of patients with anorexia nervosa [24]. Grave medical danger may suffice to trump this concern in severe anorexia nervosa.

## 6. Role of the Registered Dietitian

It is the position of the American Dietetic Association that “nutrition intervention, including nutritional counseling, by a registered dietitian (RD) is an essential component of the team treatment of patients with anorexia nervosa, bulimia nervosa and other eating disorders during assessment and treatment across the continuum of care” [25]. The role of the RD includes close monitoring and assistance in individualized meal planning and counseling to achieve successful weight restoration. In cases of refractory anorectic patients that require enteral and parenteral nutrition, the RD plays an important role in determining and monitoring the regimen and nutrition goals for these patients. 

 Daily caloric increases are a challenge for anorectic patients. Patients struggle with wanting to maintain control over their food choices and also find difficulty in making basic menu selections to meet their calorie goals. This requires a balance of patience, empathy and flexibility from the RD. It is imperative to acknowledge small successes and provide encouragement for these patients. Patients often exhibit self defeating behaviors and habits, therefore requiring consistent guidance. For example, guidelines and limitations may need to be set for the amount of time spent with the RD, menu substitutions, meal times, and meal supervision. Along with encouragement and education regarding meal plans and general nutrition, the RD can help the patient achieve goals to incorporate nutrition practices to return to a normal healthy lifestyle.

## 7. Summary

Although nutritional support of the malnourished patient is a most important component of the patient's care plan, it can worsen the patient's condition if it is used injudiciously. Because rapid refeeding of these patients can result in a catastrophic sequence of events, caution and restraint must be used when initiating therapy, together with careful monitoring of blood chemistry values and the patient's pattern and rate of weight gain. A well-balanced and nutritional oral diet plan is generally the optimal approach for refeeding these malnourished patients, but alternative modes also have a limited role.

## Figures and Tables

**Box 1:**
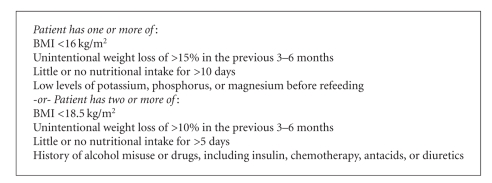
NICE guidelines for identification of patients at high risk for refeeding syndrome.

**Box 2:**
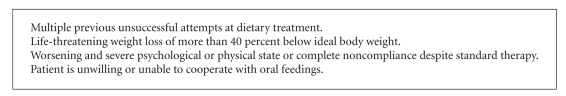
Potential indications for TPN or enteral feeding.

## References

[1] Yates A, Edman J, Aruguete M (2004). Ethnic differences in BMI and body/self-dissatisfaction among Whites, Asian subgroups, Pacific Islanders, and African-Americans. *Journal of Adolescent Health*.

[2] Castro J, Gila A, Puig J, Rodriguez S, Toro J (2004). Predictors of rehospitalization after total weight recovery in adolescents with anorexia nervosa. *International Journal of Eating Disorders*.

[3] Wiseman CV, Sunday SR, Klapper F, Harris WA, Halmi KA (2001). Changing patterns of hospitalization in eating disorder patients. *International Journal of Eating Disorders*.

[4] National Institute for Health and Clinical Excellence (2009). *Guideline for the Management of Refeeding Syndrome (Adults)*.

[5] Solomon SM, Kirby DF (1990). The refeeding syndrome: a review. *Journal of Parenteral and Enteral Nutrition*.

[6] Bermudez O, Beightol S (2004). What is refeeding syndrome?. *Eating Disorders*.

[7] Goldberg SJ, Comerci GD, Feldman L (1988). Cardiac output and regional myocardial contraction in anorexia nervosa. *Journal of Adolescent Health Care*.

[8] Gaudiani JL, Chu ES, Mehler PS (2009). Clinical issues encountered in the refeeding of the patient with anorexia nervosa. *Current Nutrition and Food Science*.

[9] Cuerda C, Ruiz A, Velasco C, Bretón I, Camblor M, García-Peris P (2007). How accurate are predictive formulas calculating energy expenditure in adolescent patients with anorexia nervosa?. *Clinical Nutrition*.

[10] Van Wymelbeke V, Brondel L, Marcel Brun J, Rigaud D (2004). Factors associated with the increase in resting energy expenditure during refeeding in malnourished anorexia nervosa patients. *American Journal of Clinical Nutrition*.

[11] Forman-Hoffman VL, Ruffin T, Schultz SK (2006). Basal metabolic rate in anorexia nervosa patients: using appropriate predictive equations during the refeeding process. *Annals of Clinical Psychiatry*.

[12] Salisbury JJ, Levine AS, Crow SJ, Mitchell JE (1995). Refeeding, metabolic rate, and weight gain in anorexia nervosa: a review. *International Journal of Eating Disorders*.

[13] Yucel B, Ozbey N, Polat A, Yager J (2005). Weight fluctuations during early refeeding period in anorexia nervosa: case reports. *International Journal of Eating Disorders*.

[14] Ornstein RM, Golden NH, Jacobson MS, Shenker IR (2003). Hypophosphatemia during nutritional rehabilitation in anorexia nervosa: implications for refeeding and monitoring. *Journal of Adolescent Health*.

[15] Mehanna HM, Moledina J, Travis J (2008). Refeeding syndrome: what it is, and how to prevent and treat it. *British Medical Journal*.

[16] Stanga Z, Brunner A, Leuenberger M (2008). Nutrition in clinical practice—the refeeding syndrome: illustrative cases and guidelines for prevention and treatment. *European Journal of Clinical Nutrition*.

[17] Patel AS, Matthews L, Bruce-Jones W (2008). Central pontine myelinolysis as a complication of refeeding syndrome in a patient with anorexia nervosa. *Journal of Neuropsychiatry and Clinical Neurosciences*.

[18] Mehler PS (2008). Use of total parenteral nutrition in severe anorexia nervosa complicated by a gastrointestinal illness. *Current Nutrition and Food Science*.

[19] Diamanti A, Basso MS, Castro M (2008). Clinical efficacy and safety of parenteral nutrition in adolescent girls with anorexia nervosa. *Journal of Adolescent Health*.

[20] Marik PE, Zaloga GP (2004). Meta-analysis of parenteral-nutrition versus enteral nutrition in patients with acute pancreatitis. *British Medical Journal*.

[21] Mehler PS, Weiner KL (2007). Use of total parenteral nutrition in the refeeding of selected patients with severe anorexia nervosa. *International Journal of Eating Disorders*.

[22] Mehler PS, Weiner KL (1993). Anorexia nervosa and total parenteral nutrition. *International Journal of Eating Disorders*.

[23] Beumont P, Carney T (2004). Can psychiatric terminology be translated into legal regulation? The anorexia nervosa example. *Australian and New Zealand Journal of Psychiatry*.

[24] Silver TJ, Robb AS, Orrell-Valente JK, Ellis N, Valadez-Meltzer A, Dadson MJ (2004). Nocturnal nasogastric refeeding for hospitalized adolescent boys with anorexia nervosa. *Journal of Developmental and Behavioral Pediatrics*.

[25] American Dietetic Association (2006). Position of the American Dietetic Association: nutrition intervention in the treatment of anorexia nervosa, bulimia nervosa, and other eating disorders. *Journal of the American Dietetic Association*.

